# IGFBP7 and the Tumor Immune Landscape: A Novel Target for Immunotherapy in Bladder Cancer

**DOI:** 10.3389/fimmu.2022.898493

**Published:** 2022-06-23

**Authors:** Xianyanling Yi, Xiaonan Zheng, Hang Xu, Jin Li, Tianyi Zhang, Peng Ge, Dazhou Liao, Hong Li, Xiaoyan Lyu, Jianzhong Ai

**Affiliations:** ^1^ Department of Urology, Institute of Urology, West China Hospital, Sichuan University, Chengdu, China; ^2^ Department of Endocrinology and Metabolism, West China Hospital of Sichuan University, Chengdu, China; ^3^ Department of Dermatology, West China Hospital, Sichuan University, Chengdu, China; ^4^ Laboratory of Dermatology, Clinical Institute of Inflammation and Immunology, Frontiers Science Center for Disease-related Molecular Network, West China Hospital, Sichuan University, Chengdu, China

**Keywords:** IFGBP7, bladder cancer, immunotherapy, tumor microenvironment, molecular subtype, hypoxia, risk model

## Abstract

Insulin-like growth factor binding protein-7 (IGFBP7) was recently reported to be a ligand of CD93, a potential target to normalize vasculature and attenuate immunotherapy. However, its role in the tumor microenvironment (TME) and immunotherapy response of bladder cancer (BLCA) remains unclear. We comprehensively evaluated the correlation between IGFBP7 and multiple immunological characteristics of BLCA across The Cancer Genome Atlas (TCGA) and two external cohorts. Importantly, the response of IGFBP7-grouped BLCA patients to immunotherapy was predicted and validated by five real-word immunotherapy cohorts. Finally, we developed an IGFBP7-based immune risk model validated by five independent cohorts. IGFBP7 modulated the TME across pan-caners. In BLCA, high expression of IGFBP7 was correlated with more aggressive clinical features. IGFBP7 was positively associated with immunomodulators and promoted tumor-infiltrating lymphocyte trafficking into the tumor microenvironment. However, T cells recognition and tumor cell killing were lower in the high-IGFBP7 group. In addition, high expression of IGFBP7 displayed lower enrichment scores for most pro-immunotherapy pathways. Clinical data from IMvigor210 and GSE176307 indicated that IGFBP7 negatively correlated with the BLCA immunotherapy response. The same trend was also observed in a renal cell carcinoma (RCC) cohort and two melanoma cohorts. Notably, urothelial and luminal differentiation were less frequently observed in the high-IGFBP7 group, while neuroendocrine differentiation was more frequently observed. Mechanistically, high IGFBP7 was associated with an enriched hypoxia pathway and higher expression of key genes in ERBB therapy and antiangiogenic therapy. Furthermore, our IGFBP7-based immune risk model was able to predict the prognosis and response to immunotherapy with good accuracy (5-year AUC = 0.734). Overall, IGFBP7 plays a critical role in the immunoregulation and TME of BLCA and may serve as a novel potential target for combination treatment with immunotherapy for BLCA.

## Introduction

Bladder cancer (BLCA) is one of the most common malignancies of the urinary system, with approximately 212,536 deaths each year from BLCA (1). Generally, the treatment strategies of BLCA include surgical resection, radiation therapy, chemotherapy and immunotherapy (2, 3). Some patients with local BLCA can be curable; however, the five-year survival rate of patients with metastatic bladder cancer is low (4). In recent years, significant advantages have been made in terms of immunotherapy and targeted therapies for bladder cancer (5). Immune checkpoint inhibitors (ICIs), as one of the most promising types in immunotherapy, are widely used in treating different kinds of cancers (6, 7). ICIs have also been reported to be relatively effective for BLCA (8, 9), which may be attributed to the high tumor mutation burden and abundant infiltration of immune cells within the tumor microenvironment (TME) of BLCA (10–12). Five ICIs have been approved for the treatment of locally advanced and metastatic BLCA by the FDA (13). However, the response to ICIs varies across BLCA patients, and only a minority of BLCA patients benefit from these agents (14, 15). To date, there is still a lack of novel drugs for the development of more effective therapeutic strategies.

Insulin-like growth factor binding protein-7 (IGFBP7) is a member of the IGFBP family, which binds insulin with high affinity and IGF with low affinity (16). IGFBP7 was originally identified in normal mammary epithelial cells and meningeal cells, and its expression pattern varies with tumor type (17). In some tumors, IGFBP7 exhibits tumor suppressor activity in certain cancer types *via* regulation of cell proliferation, apoptosis, cell adhesion epithelial mesenchymal transition (EMT) and angiogenesis (18–20). However, IGFBP7 acts as a cancer-promoting gene in esophageal adenocarcinoma and neck squamous cell carcinomas (21, 22). Recently, Sun et al. (23) found that IGFBP7, acting as a ligand of CD93, can disrupt normalizes tumor vasculature and increase immune infiltration through the CD93/IGFBP7 pathway. Moreover, a CD93-targeting monoclonal antibody (mAb) has been demonstrated to reduce tumor growth and enhance the effects of immunotherapy in pancreatic tumors or melanoma *via* the CD93/IGFBP7 pathway (24). Together, IGFBP7 may provide potential value for the immunotherapy of cancer, but the role of IGFBP7 in BLCA has not been elucidated.

In this study, IGFBP7 was highly correlated with the modulation of the TME in most cancers by pan-cancer analysis. We found that IGFBP7 was negatively associated with T cells recognition and tumor cell killing. In addition, IGFBP7 negatively correlated with the BLCA immunotherapy response, and IGFBP7 had the potential to predict the molecular subtype of BLCA. Anti-IGFBP7 therapy may be a suitable therapeutic candidate for BLCA, but more studies are required for further validation.

## Materials and Methods

### Data Source and Preprocessing

The mRNA sequencing expression profiles and clinical information of bladder cancer patients were downloaded from The Cancer Genome Atlas (TCGA) (http://cancergenome.nih.gov/). The abbreviations for various cancer types are listed in Table S1. The advanced urothelial cancer cohort (IMvigor210 cohort) (patients treated with atezolizumab) based on the Creative Commons 3.0 License was downloaded from a freely available data package (http://research-pub.gene.com/IMvigor210CoreBiologies/) (25). Five BLCA datasets (GSE176307, GSE13507, GSE31684, GSE32894, GSE48277) and two melanoma datasets (GSE78220, GSE91061) were downloaded from the Gene Expression Omnibus (GEO) database (https://www.ncbi.nlm.nih.gov/geo/). Of these cohorts, GSE176307 (patients treated with pembrolizumab or atezolizumab), GSE78220 (patients treated with pembrolizumab and nivolumab) (26), and GSE91061 (patients treated with nivolumab) (27) were all treated with immunotherapy. In addition, another immunotherapy cohort of renal cell carcinoma (RCC) (PMID29301960) (patient treated with nivolumab) was obtained from Miao’s study (28). The raw data are shown in **Supplementary Material**.

### Pan-Cancer Analysis of IGFBP7

For the pan-cancer analysis, the R package “ggplot2” was used to identify the Spearman correlations between the expression of IGFBP7 and immunomodulators, which include immunostimulators, chemokines, major histocompatibility complex (MHC) and receptors (29). Correlations between the expression of IGFBP7 and immune checkpoints (CD274, CTLA4, HAVCR2, LAG3, PDCD1, PDCDLIG2, TIGIT, and SIGLEC15) were also computed. IGBP7 expression was measured in tumor-infiltrating immune cells (B cells, CD4+ T cells, CD8+ T cells, neutrophils, macrophages, and dendritic cells) by using TIMER 2.0 (http://timer.cistrome.org/) (30).

### Association Between IGFBP7 Expression and Clinical Features

The patients were grouped based on different clinical features, including T stage (T1&T2 versus (vs) T3&T4), N stage (N0 vs N+), lymphovascular invasion (yes vs no), pathologic stage (stage I&II vs stage III&IV), histologic grade (low grade vs high grade) and histologic subtype (papillary vs non-papillary). According to the results of the normality test and homogeneity of variances, independent samples t tests were used to evaluate the differential expression of IGFBP7 between different groups.

### The Effect of IGBP7 on Immunological Characteristics in BLCA

Coexpression was analyzed statistically by using the Spearman correlation coefficient to identify the expression differences of 122 immunostimulators between the high- and low-IGFBP7 groups (The patients in the same cohort were divided into high and low subgroups based on the median IGFBP7 expression value). Single-sample gene set enrichment analysis (ssGSEA) was used to quantify the relative abundance of 15 immune cell infiltrates (31). Subsequently, the infiltration of immune cells was compared between the high- and low-IGFBP7 groups using the Wilcoxon rank sum test. The R package “ComplexHeatmap” was used to visualize the expression of genes frequently expressed on the surfaces of immune cells (32). The cancer immunity cycle consists of the following seven steps: release of cancer cell antigens (Step 1), cancer antigen presentation (Step 2), priming and activation (Step 3), trafficking of immune cells to tumors (Step 4), infiltration of immune cells into tumors (Step 5), recognition of cancer cells by T cells (Step 6), and killing of cancer cells (Step 7). Tracking tumor immunophenotype (TIP, http://biocc.hrbmu.edu.cn/TIP/) was used to analyze and visualize the cancer immunity cycle (33). The status of anti-cancer immunity was compared according to IGFBP7 groups, and we plotted a heatmap with the R package ComplexHeatmap. Pan-cancer T cell-inflamed score can also define pre-existing cancer immunity, which includes eighteen genes (34). Moreover, 18 inhibitory immune checkpoints with therapeutic potential were selected, and then the correlations between them and IGFBP7 were assessed.

### Prediction of Immunotherapeutic and Chemotherapy Drug Response

We compared the different expression levels of eight immune checkpoint-related genes between the high- and low-IGFBP7 groups. Eighteen immunotherapy-positive signatures were included in our study, and their enrichment scores were calculated using gene set variation analysis (GSVA). Comparisons for predicting the response to immunotherapy were performed between the high- and low-IGFBP7 groups. The TIDE algorithm was used to predict potential ICB responses in the high- and low-IGFBP7 groups (35). The immunotherapy response data for two BLCA cohorts (IMvigor210 and GSE176307), RCC cohort (PMID29301960) and two melanoma cohorts (GSE78220 and GSE91061) were collected. We evaluated IGFBP7 expression in the PR/CR group and SD/PD group. In addition, tumor mutation burden was compared in the high- and low-IGFBP7 groups. Moreover, we predicted IGFBP7-grouped chemotherapy and tyrosine kinase inhibitor drug responses based on the Genomics of Drug Sensitivity in Cancer (GDSC) database (https://www.cancerrxgene.org/) (36). The prediction process was implemented by the R package “pRRophetic”, where the samples’ half-maximal inhibitory concentration (IC50) was estimated by ridge regression and the prediction accuracy.

### Enrichment Analysis of Various Therapeutic Signatures and BLCA Molecular Subtypes

By using consensus MIBC and BLCA subtyping R packages, the samples were assigned to different BLCA molecular subtypes, which included a combined consensus subtype and six published molecular classifications (University of North Carolina (UNC), Baylor, Cancer Genome Atlas (TCGA), MD Anderson Cancer Center (MDA), Lund and Cartes d’Identité des Tumeurs (CIT)) (37–42). Afterwards, we calculated the enrichment score of the 12 molecular subtype-specific signatures for the training and validation cohorts (37). A gene set enrichment analysis was performed computed by GSVA to evaluate various therapeutic signatures in both the training cohort and validation cohort. The results are presented in the form of a heatmap, as well as bar graphs.

### Identification of Gene Mutation Analysis and Drug-Related Genes of BLCA

To identify somatic mutations in patients with BLCA in the TCGA database, mutation data were retrieved from the TCGA database and visualized using the “maftools” package in R software. The waterfall plot shows the mutation data of the top 30 mutated genes. We further used the data of BLCA-related drug target genes obtained from the DrugBank database (https://go.drugbank.com/) to compare their expression in IGFBP7 groups (43).

### Development of an IGFBP7-Based Immune Risk Model

We selected the dataset from TCGA as the training cohort. The DESeq2 R package was used to analyze the differentially expressed genes (DEGs) in the high- and low-IGFBP7 groups. Prognostic genes of TCGA-BLCA were screened with a P value < 0.05. Immunologic signature gene sets were downloaded from The Molecular Signatures Database (MSigDB) C7 dataset (https://www.gsea-msigdb.org/gsea/msigdb/index.jsp). Next, the three gene sets obtained were intersected and served as the candidate gene set. The least absolute shrinkage and selection operator (LASSO) Cox regression method using the “glmnet” and “survival” R packages was applied to select the optimal corresponding coefficients for risk model construction. Based on the following formula, the risk score for each patient was calculated.


$$\text{RiskScor}{\text{e}}_{i}=\sum_{j=1}^{n}exp_{ji}\times \beta_{j}$$

where *exp* means the gene expression value, *i* means each sample, *j* means each gene, and *β* means the coefficient in LASSO regression. A forest plot was used to explore the correlation between the genes and prognosis in BLCA. A Kaplan–Meier curve was drawn to compare the overall survival between the high-risk and low-risk groups. Receiver operating characteristic (ROC) curve analyses and decision curve analyses (DCA) were conducted to evaluate the model. For validation of the risk model, five independent cohorts (GSE13507, GSE31684, GSE32894, GSE48277, IMvigor210) were used. Furthermore, we explored the associations between the risk model and clinicopathological features, tumor microenvironment features, various therapeutic signatures, immune checkpoint genes, BLCA molecular subtype and BLCA-related drug target genes.

### Statistical Analysis

All statistical data analyses were performed using R software, version 3.6.3. Continuous variables that conformed to the normal distribution were compared using independent t tests for comparisons between binary groups, while continuous variables with skewed distributions were compared with the Mann–Whitney U test. Categorical variables were compared by using the chi-square test or Fisher’s exact test. Spearman analysis was used for the correlation studies between quantitative variables. Survival curves were analyzed using the log-rank test (generated using the Kaplan–Meier method). All statistical tests were two-sided with a level of significance set as P < 0.05.

## Result

### The Immunological Role of IGFBP7 in Pan-Cancer

**Figure 1:**
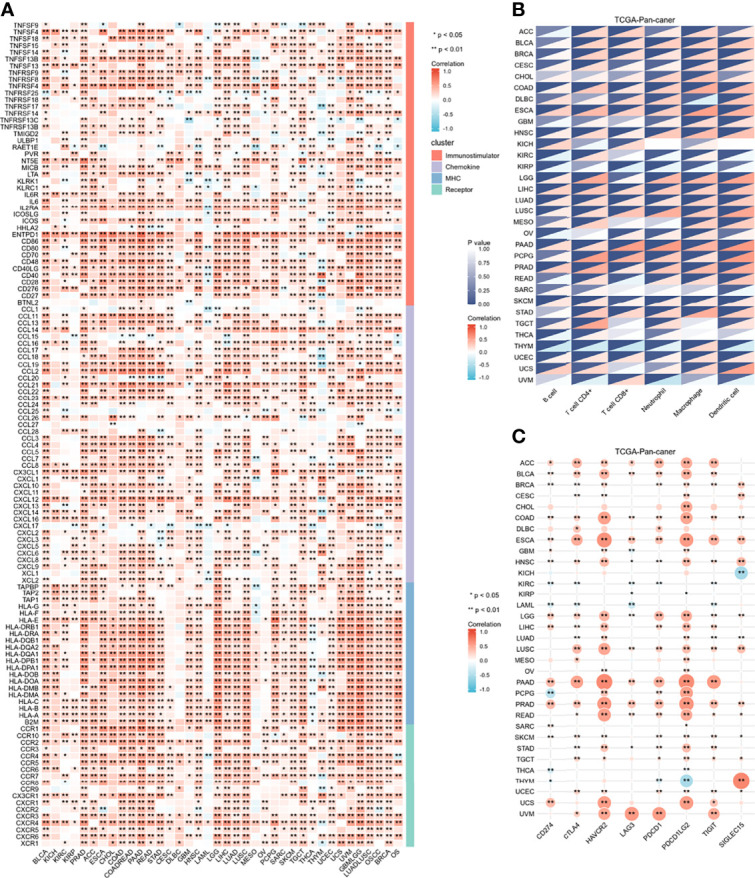
Immunological role of IGFBP7 in Pan-cancer. **(A)** correlations between the expression of IGFBP7 and immunomodulators (immunostimulatory, chemokine, MHC, receptor). **(B)** correlations between the expression of IGFBP7 and tumor infiltrating immune cells. **(C)** correlations between the expression of IGFBP7 and immune check point. The value of the correlation coefficient is represented by the intensity of blue or red, as indicated on the color scale. The asterisks indicate significant differences calculated using spearman correlation analysis. (*p < 0.05; **p < 0.01).

To determine the role of IGFBP7 in regulating the microenvironmental immunity of cancer, correlations between the expression of IGFBP7 and immunomodulators, immune checkpoints and tumor-infiltrating immune cells were performed. The results demonstrated that IGFBP7 was positively correlated with a majority of immunomodulators in the majority of cancers (**Figure 1A**). We also calculated the infiltration levels of TIICs in the TME using the ssGSEA algorithm. Except for KICH, KIRC, KIRP, SARC, TGCT, THCA, THYM and UVM, IGFBP7 exhibited a positive correlation with the majority of TIICs in most of the cancer types (**Figure 1B**). Additionally, we found that the expression of IGFBP7 was mutually exclusive to immune checkpoints in THCA, THYM, LAML, KIRC and KIRP. IGFBP7 was positively related to most immune checkpoints in other malignancies (**Figure 1C**). Overall, these findings indicated that IGFBP7 played a key role in regulating microenvironment immunity across most cancers.

### Clinical Relevance of IGFBP7

**Figure 2:**
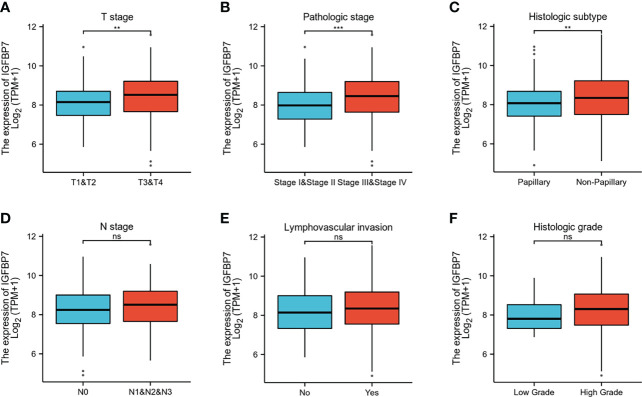
IGFBP7 expression and clinicopathological features in BLCA. **(A–C)** High T stage, high pathologic stage and non-papillary subtype were associated with higher expressions of IGFBP7 in BLCA. **(D–F)** No statistically significant differences were found between the expression levels of IGFBP7 in BLCA and N stage, lymphovascular invasion and histologic grade. **p < 0.01; ***p < 0.001; ns, no significance.

The gene expression profiling data and clinical information of BLCA patients were downloaded from the TCGA database. The patients with BLCA were divided into different groups based on clinical parameters to analyze differences in gene expression. High expression of IGFBP7 was significantly related to advanced T stage, pathologic stage and poorly differentiated histologic subtype (**Figures 2A–C**). There were no statistically significant differences in the pattern of gene expression between N stage, lymphovascular invasion and histologic grade (**Figures 2D–F**).

### The Immunological Role of IGFBP7 in the TME of BLCA

**Figure 3:**
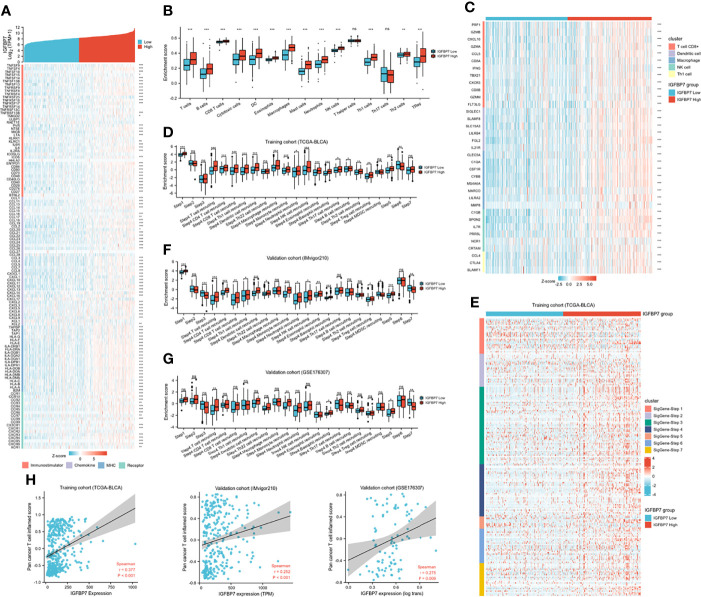
The effect of IGBP7 on Immunological Characteristics in BLCA. **(A)** Differences in the expression of 122 immunomodulators (immunostimulators, chemokines, MHC and receptors) between high- and low-IGFBP7 groups in BLCA. **(B)** Enrichment scores of 15 immune cell infiltrates in high- and low-IGFBP7 groups. **(C)** Expression levels of the gene markers of the five common TIICs in the high- and low-IGFBP7 groups. **(D, F, G)** The activities of the various steps of the cancer immunity cycle calculated by ssGSEA algorithm in the high- and low-IGFBP7 groups in three cohorts. **(E)** Differences in the various steps of the cancer immunity cycle between high- and low-IGFBP7 groups. **(H)** Correlations between IGFBP7 and the pan-cancer T cell inflamed score in the TCGA, IMvigor210 and GSE176307 cohorts. *p< 0.05; **p<0.01; ***p<0.001. ns, no significance; ns, p≥0.05.

Based on the above pan-caner analyses, IGFBP7 showed a strong correlation with immunomodulators. We further examined the association between immunomodulators and the expression level of IGFBP7 (**Figure 3A**). This result indicated that IGFBP7 was significantly positively correlated with a majority of immunostimulators. Chemokines contributed to recruitment of CD8+ T cells, macrophages, TH17 cells, and antigen-presenting cells were also upregulated in the IGFBP7 high group. The results for major histocompatibility complex (MHC) and receptors demonstrated the same trend.

To further understand and characterize the microenvironment immunity with IGFBP7 expression, the profile of TME cell infiltration models was evaluated. We found that high expression of IGFBP7 prompted immune cell infiltration in the TME, with the exception of T helper cells and T17 cells (**Figure 3B**). The infiltration of regulatory T cells (Tregs), which have immunosuppressive effects, was also enhanced in the high-IGFBP7 group. Likewise, IGFBP7 was positively correlated with the effector genes of these TIICs (**Figure 3C**). Moreover, as a direct systematic performance of the functions of the chemokines and other immunomodulators, the activities of the cancer immunity cycle (**Figure 3D**), including the release of cancer cell antigens (Step 1), trafficking of immune cells to tumors (Step 4), and infiltration of immune cells into tumors (Step 5), were found to be upregulated in the IGFBP7 high group. Notably, the recognition of cancer cells by T cells (Step 6) was weakened by IGFBP7. These results were also presented by the heat-map graph (**Figure 3E**), and they are consistent with previous findings. Validation cohorts (**Figures 3F, G**) showed the same trend in some steps (Step 1, Step 4 and Step 5) of cancer immunity cycle. Although we did not observe that high expression of IGFBP7 could significantly weaken the Step 6, in these two validation cohorts, the killing of cancer cells (Step 7) was downregulated in the IGFBP7 high group. Collectively, IGFBP7 shaped a hostile TME. The results of the correlation between IGFBP7 gene expression and the T-cell inflamed ssGSEA score indicated that IGFBP7 expression was significantly positively related to the pan-cancer T cell inflamed score in the training cohort and validation cohorts (IMvigor210 cohort and GSE176307) (**Figure 3H**). Furthermore, the inflamed TME exhibited higher immune checkpoint inhibitor expression levels (44). Consistently, IGFBP7 had positive correlations with the vast majority of inhibitory immune checkpoints (**Supplementary Figure S1**).

### IGFBP7 Predicts Immunotherapy Response in BLCA

**Figure 4:**
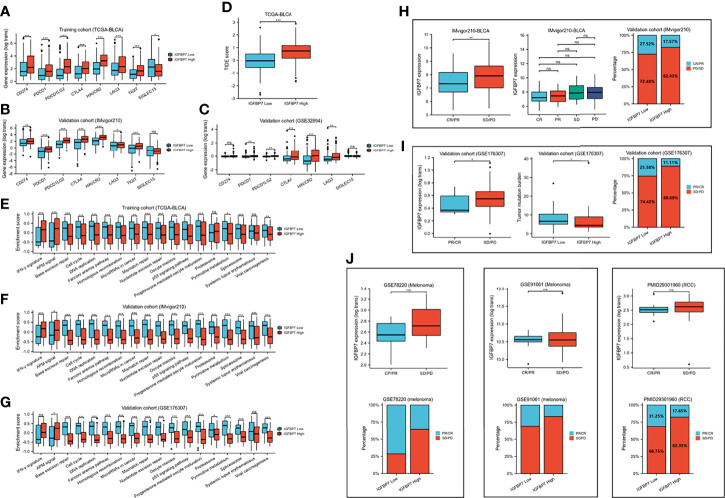
IGFBP7 predicts therapeutic response to immunotherapy in BLCA. **(A–C)** Expression levels of immune check points in the high- and low-IGFBP7 groups in the TCGA, IMvigor210, GSE32894 cohorts. **(D)** ICB responses in the high- and low-IGFBP7 groups using TIDE algorithm. **(E–G)** The enrichment scores of several immune-related signatures in the high- and low-IGFBP7 groups. **(H, I)** Correlation between IGFBP7 and the clinical response of cancer immunotherapy in the IMvigor210 and GSE176307 cohort. **(J)** Correlation between IGFBP7 and the clinical response of cancer immunotherapy in the RCC cohort and two melanoma cohorts. CR: complete response; PR, partial response; PD, progressed disease; SD, stable disease. (CR/PR means patient who are CR or PR; SD/PD means patient who are SD or PD). *p< 0.05; **p<0.01; ***p<0.001. ns, no significance; ns, p≥0.05.

We compared the expression of several common immunotargets, including CD274, PDCD1, PDCD1LG2, CTLA4, HAVCR2, LAG3, TIGT and SIGLEC15, between the high- and low-IGFBP7 expression subgroups by using the training and validation cohorts, and the results showed that the expression of immunotargets was higher in the IGFBP7 high group (**Figures 4A–C**). As expected, a higher TIDE score occurred in the IGFBP7 high group (**Figure 4D**), which indicated that the IGFBP7 high group showed worse clinical efficacy to ICB therapy. In addition, IGFBP7 negatively correlated with the enrichment scores of most immunotherapy-positive gene signatures in the TCGA, IMvigor210 and GSE176307 cohorts (**Figures 4E–G**).

Subsequently, we collected immunotherapy response data for IMvigor210 and GSE176307 and evaluated IGFBP7 expression in the PR/CR group and SD/PD group. In line with the results of the response to ICB, IGFBP7 was shown to have lower expression in the PR/CR group than in the SD/PD group (**Figures 4H, I**). The same trend occurred in three external cohorts (RCC cohort and two melanoma cohorts) (**Figure 4J**). However, there were no significant differences. The tumor mutation burden was significantly higher in the IGFBP7 low group (**Figure 4I**).

### IGFBP7 Predicts the Response to Chemotherapy Drugs and Tyrosine Kinase Inhibitors in BLCA

**Figure 5:**
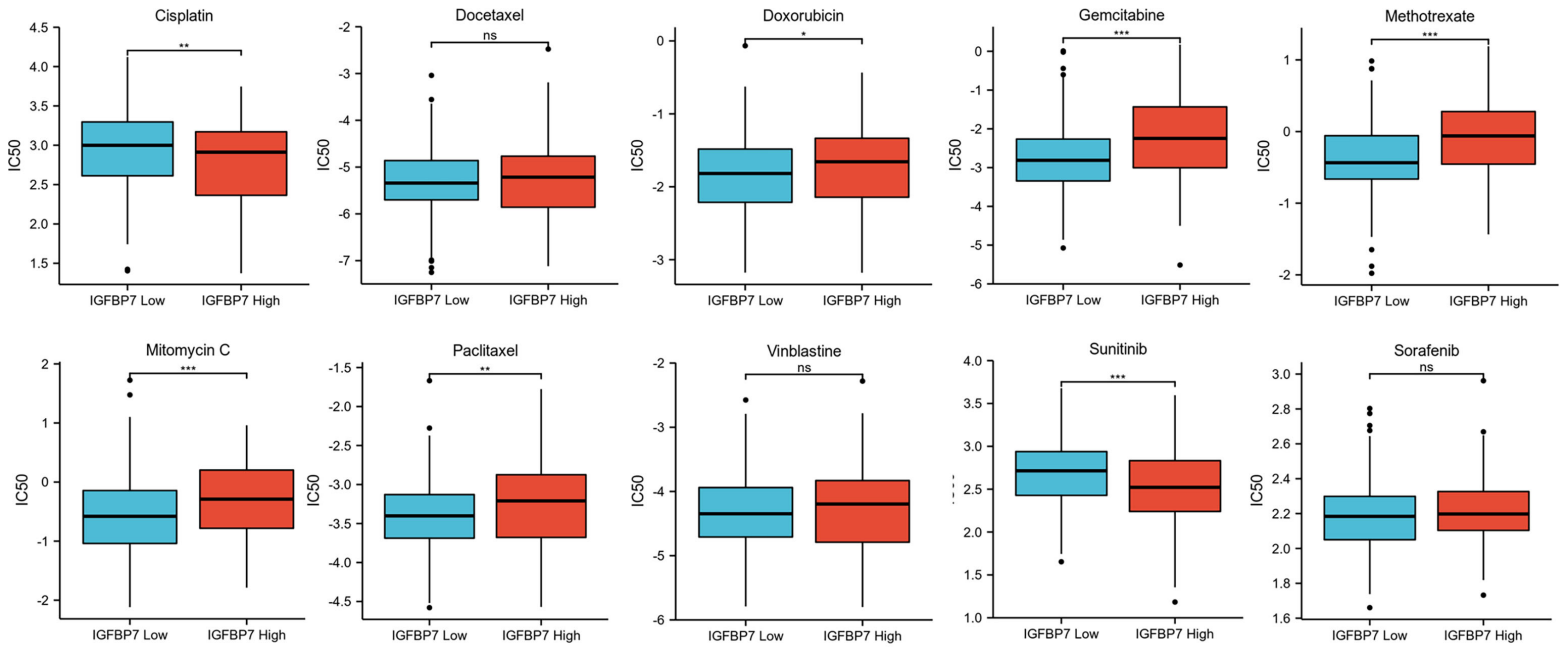
IC50 of chemotherapy drugs and tyrosine kinase inhibitors in bladder cancer based on IGFBP7 expression. *p< 0.05; **p<0.01; ***,p<0.001. ns, no significance; ns, p≥0.05.

We predicted the response to chemotherapy drugs and tyrosine kinase inhibitors for the high- and low-IGFBP7 groups based on the DrugBank database. The results indicated that patients with low expression of IGFBP7 were more sensitive to doxorubicin, gemcitabine, methotrexate, mitomycin C and paclitaxel (**Figure 5**). Nevertheless, a significantly higher response to cisplatin and sunitinib was observed in the IGFBP7 high group.

### IGFBP7 Predicts Molecular Subtypes and Therapeutic Opportunities in BLCA

**Figure 6:**
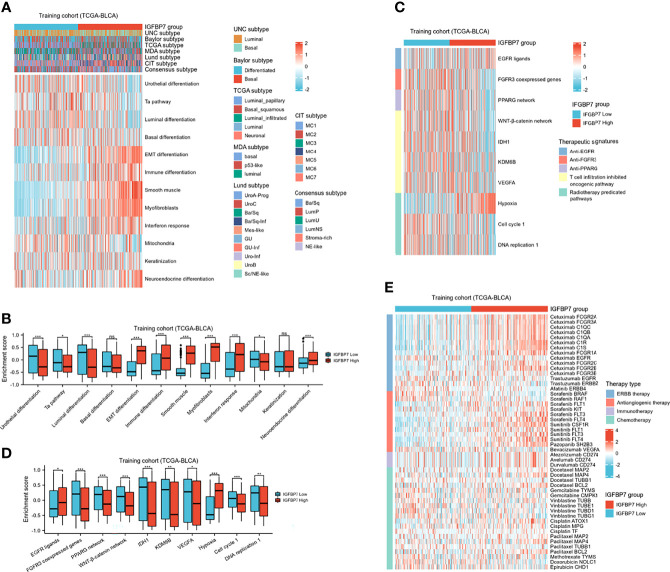
IGFBP7 predicts the molecular subtype and the therapeutic response to several therapies in BLCA. **(A)** Correlations between IGFBP7 and molecular subtypes using different algorithms and bladder cancer signatures. **(B)** The enrichment scores of 12 molecular subtype-specific signature in the high- and low-IGFBP7 groups. **(C, D)** Correlations between IGFBP7 and the enrichment scores of several therapeutic signatures. **(E)** Correlation between IGFBP7 and the BLCA-related drug-target genes obtained from the Drugbank database. *p< 0.05; **p<0.01; ***p<0.001. ns, no significance; ns, p≥0.05.

BLCA molecular typing was conducted in multiple research centers and named the UNC subtype, Baylor subtype, TCGA subtype, MDA subtype, Lund subtype, CIT subtype and consensus subtype. Despite these variations, all typing methods contain two fundamental subtypes: the basal subtype and the luminal subtype (45). The basal subtype has a poorer prognosis than the luminal subtype but is neo-adjuvant chemotherapy (NAC)-sensitive (46, 47). To further explore the expression patterns of IGFBP7 in BLCA, we evaluated the distribution of IGFBP7 in different molecular subtypes. In the training cohort, we found IGFBP7 to be negatively related to the luminal differentiation subtype of BLCA (**Figure 6A**). In addition, the enrichment scores for urothelial differentiation, the Ta pathway, luminal differentiation and mitochondria were greater in the low-IGFBP7 group. The enrichment scores for EMT differentiation, immune differentiation, smooth muscle, myofibroblast, interferon response and neuroendocrine differentiation were higher in the high-IGFBP7 group (**Figure 6B**). We validated these outcomes by using two external cohorts (**Supplementary Figure S2A–D**).

In addition, we performed enrichment analysis to evaluate various therapeutic signatures in different IGFBP7 groups. The difference in enrichment scores for various therapeutic signatures between the high- and low-IGFBP7 groups was significant (**Figure 6C, D**). Notably, the enrichment scores for hypoxia were lower in the low-IGFBP7 group, which was not the same as other radiotherapy-predicted pathways. IMvigor210 and GSE176307 were used to validate our outcomes (**Supplementary Figures S2E–H**). Moreover, the results from the DrugBank database indicated a notably higher response to ERBB therapy and antiangiogenic therapy in the high-IGFBP7 group (**Figure 6E**; **Supplementary Figures S2I, J**). We further visualized the mutation data of TCGA-BLCA, and the top 30 mutated genes are displayed (**Supplementary Figure 3A**). Likewise, tumor mutation burden was calculated and compared between the high- and low-IGFBP7 groups. There were no significant differences between the high- and low-IGFBP7 groups (**Supplementary Figure 3B**).

### Development and Validation of the IGFBP7-Based Immune Risk Model

**Figure 7:**
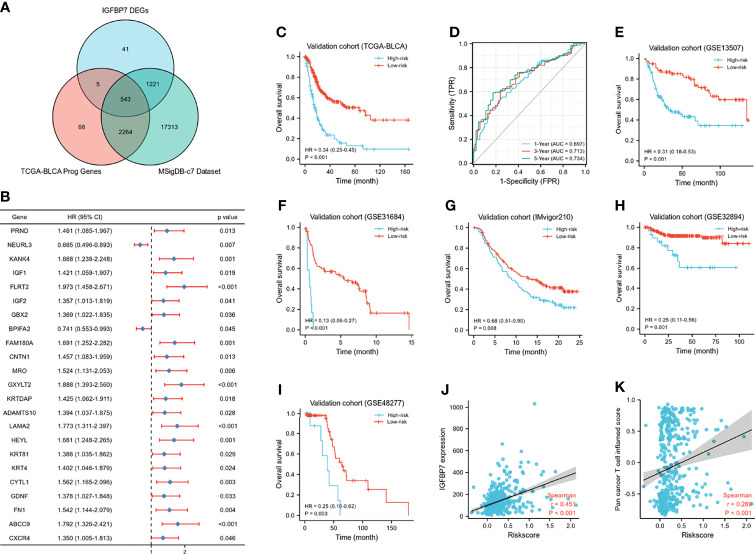
Development of IGFBP7-based immune risk model. **(A)** Venn diagram showing the candidate gene set. **(B)** Forest plot of the correlation between the candidate genes and prognosis in BLCA. **(C, D)** Kaplan-Meier survival curve analysis of the low- and high-risk in TCGA training set and the predictive accuracy of risk model for survival. **(E–I)** Validation of the risk model in five external independent sets: GSE13507, GSE31684, GSE32894, GSE48277, IMvigor210. **(J, K)** Correlations between riskscore, expression of IGFBP7 and pan-cancer T cell inflamed score.

**Figure 8:**
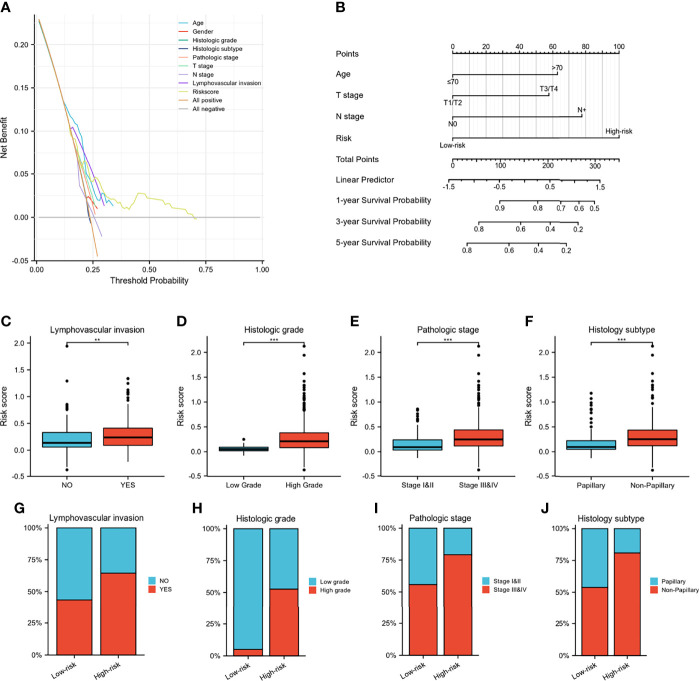
Validation of IGFBP7-based immune risk model. **(A)** DCA curve for assessment of the clinical usefulness of the risk model. **(B)** Nomogram for predicting the probability of 1-, 3-, and 5-year survival probability for BLCA patients. **(C–J)** Differences in clinicopathological features between the high- and low-risk groups in the TCGA cohort. **p<0.01; ***p<0.001.

A Wayne diagram showed that 543 candidate genes were significantly related to prognosis (**Figure 7A**). Subsequently, the LASSO algorithm was used to identify the 23 best candidate genes. The forest plots illustrated univariate Cox analysis of the prognostic impact of the candidate gene set (**Figure 7B**). The risk score model was constructed based on the training cohort. ROC analysis was used to test our model, and the area under the ROC curve was above 0.697, which meant a moderate sensitivity and specificity for predicting the prognosis of BLCA (**Figure 7D**). Furthermore, we divided the patients into high- and low-risk groups based on their risk scores. As shown in **Figure 7C**, there was a significant difference in overall survival between the two groups. The risk model was then validated using another five sets of validation datasets (**Figures 7E–I**). Considering the clinical usefulness of the risk model, we drew a DCA curve. According to the DCA, when the threshold probability for a patient was within the approximate range of 20-100%, the risk model added more net benefit than the “all positive” or “all negative” strategies in the TCGA cohort (**Figure 8A**). A nomogram model was constructed for predicting the prognosis of BLCA by using clinical characteristics, including age, T stage, N stage and risk score. The nomogram plot in **Figure 8B** shows the weight of each variable based on the multivariate Cox analysis, and the straight line down to the endpoint scales could predict the probability of survival at 1, 3, and 5 years. In addition, as expected, patients with lymphovascular invasion, high histologic grade, advanced pathologic stage and non-paillary subtype were more likely to obtain a higher risk score (**Figures 8C–J**).

Except for the value of predicting prognosis, our risk model significantly predicted the response to immunologic therapy. The expression of IGFBP7 and pan-cancer T cell inflamed score were both significantly positively correlated with the risk score (**Figures 7J, K**). Furthermore, the enrichment scores of most of the cancer immunity cycle steps were higher in the high-risk group (**Supplementary Figure S4A**). Similarly, the expression of a majority of immune checkpoints was higher in the high-risk group (**Supplementary Figure S4B**). The association between the risk score and different molecular subtypes was in line with previous findings. Patients with basal-type bladder cancer had a higher risk score and a worse prognosis (**Supplementary Figures S4C, D**). We also discovered that the enrichment scores of T cell infiltration inhibited oncogenic pathways were significantly higher in the high-risk group, while those in hypoxia were higher in the low-risk group (**Supplementary Figures S4E, F**). Finally, according to the heatmap, ERBB therapy, antiangiogenic therapy, immunotherapy and chemotherapy may be appropriate for BLCA with higher risk scores (**Supplementary Figures S4G**).

## Discussion

As one of the most common malignant tumors of the urinary system, BLCA lacks effective targeted treatment. With the great advances of immunotherapy in BLCA, its favorable safety and tolerability are progressively manifested. However, given that the overall response rates to immunotherapy are still low, more accurate and effective targets for immunotherapy are urgently needed. It is generally known that IGFBP7 plays a role in tumorigenesis *via* the IGF/insulin signaling pathway. IGFBP7 can block downstream signaling and impede cell growth, apoptosis and the TME. Previous studies indicated that IGFBP7 functions as a tumor suppressor in several tumors, including hepatocellular carcinoma, colorectal carcinoma, prostate cancer cells, and breast cancer (17, 48–50). Nevertheless, some differences in results were found in esophageal adenocarcinoma and neck squamous cell carcinomas (21, 22). The role of IGFBP7 in BLCA is still unclear, and more evidence is needed to explore the association between IGFBP7 and tumor immunologic features.

In this study, pan-cancer analysis indicated that IGFBP7 was positively correlated with immunomodulators, the infiltration levels of TIICs, and immune checkpoints in the majority of cancers. It is well known that the TME mediates immune escape and regulates the sensitivity of tumors to anticancer drugs. The TME is composed of various types of immune cells, including T cells, NK cells and dendritic cells, which are responsible for anticancer immunity (51). Regulatory T cells (Tregs) have immunosuppressive effects because they can promote evasion of the recognition of tumor antigens by antigen-presenting cells and T cells (52). More importantly, IGFBP7 showed a strong correlation with the TME in BLCA. We found that most immunostimulators and TIICs were significantly upregulated in the IGFBP7 high group. Additionally, the enrichment score of Tregs was also higher in the IGFBP7 high group. In the tumor immunity cycle, IGFBP7 enhanced the release of cancer cell antigens, trafficking of immune cells to tumors and infiltration of immune cells into tumors. Theoretically, high IGFBP7 expression may result in a better immune microenvironment. However, the recognition of cancer cells by T cells eventually weakened in TCGA cohort. Meanwhile, we did not observe that the killing of cancer cells was significantly downregulated by IGFBP7 in the TCGA cohort but was found in both the IMvigor 210 and GSE176307 cohorts.

Abnormal tumor blood vessels cause the formation of an immunosuppressive microenvironment, leading to immune escape. Several studies have demonstrated that IGFBP7 is typically overexpressed in tumor-associated endothelial cells relative to normal vascular endothelial cells (53–55). Sun et al. (23) demonstrated that IGFBP7 acted as a ligand of CD93 and disrupted normalizes tumor vasculature, including reducing pericyte and smooth muscle cell coverage on blood vessels and increasing vascular permeability and leakage by the CD93/IGFBP7 pathway. Abnormal tumor vascular structure and function lead to interstitial hypertension and a hostile TME characterized by hypoxia and acidosis. These changes in the tumor microenvironment may influence immune cell function. Previous studies indicated that hypoxia and acidosis hindered the maturation of APCs and DCs, and immature DCs could not activate T cells effectively, although they can still present antigens (56, 57). Although immune infiltration can increase *via* the interaction of IGFBP7 and CD93, hypoxia drives the preferential recruitment of Tregs, which express negative costimulatory molecules and lead to inadequate costimulation for T-cell activation (58, 59). In the meantime, infiltrated TILs were inactive, and effector T cells were unable to recognize and kill the tumor cells. Therefore, as we demonstrate here, high expression of IGFBP7 did not seem to enhance the cancer immunity cycle but tended to diminish. Furthermore, we offer the following conjecture regarding why higher IGFBP7 expression is accompanied by more enriched TILs: the increases in tumor vascular permeability can result in leakage through the vessel wall, which allows the tumor cells and TILs to leave the tissue more easily. However, further studies are required to verify this conjecture. Overall, high expression of IGFBP7 increased TIICs, but the activities of the recognition of cancer cells by T cells and killing of cancer cells were decreased. These factors may shape the different responses to immune therapy between patients with high and low IGFBP7 expression.

In theory, the expression of immune checkpoints will be upregulated in an immunosuppressive microenvironment, and this was indeed the case. In our further analysis, we found that high IGFBP7 expression was correlated with a lower response to ICB. This result was consistent with the views of Sun et al (24). In addition, the enrichment scores of immunotherapy-positive signatures showed that most immunotherapy-positive signature enrichment scores were higher in the IGFBP7 low group. Clinical data indicated concordant findings. We found that IGFBP7 was significantly inversely correlated with the immunotherapeutic response in two BLCA cohorts (IMvigor210 and GSE176307). The same tendency was also found for two additional melanoma cohorts and the RCC cohort. Moreover, immunosuppressive oncogenic pathways, such as the FGFR3, PPARG, and β-catenin pathways, were found to suppress the infiltration of TIICs *via* a reduction in the expression of immunomodulators (60–62). IGFBP7 was remarkably negatively correlated with these oncogenic pathways, which was consistent with our previous results. Notably, IGFBP7 expression was positively related to the enrichment scores of immunosuppressive pathways, including anti-EGFR and hypoxia therapy. In terms of chemotherapy drug response, the lower expression of IGFBP7 could make the cancer cells more sensitive to most chemotherapy drugs but resistant to cisplatin. Furthermore, IGFBP7 expression predicted the response to therapeutic options in BLCA, and it showed a notably higher response to ERBB therapy and antiangiogenic therapy in the high-IGFBP7 group.

Molecular subtype can help prognosticate and predict the response to immunotherapies, radiotherapy, neoadjuvant chemotherapy and several targeted therapies (37–39, 63). We found that high expression of IGFBP7 was less likely to be a luminal differentiation subtype, but the luminal subtype was related to a better prognosis. Moreover, the enrichment scores for urothelial differentiation, the Ta pathway, luminal differentiation and mitochondria were higher in the high-IGFBP7 group. All the results above were validated in independent cohorts. Eventually, we constructed a risk model to predict prognosis and the response to immunologic therapy. Our model was externally validated to show good robustness.

Given the above, IGFBP7 plays an important role in the regulation of the tumor microenvironment and impacts the immunotherapy response. We hypothesize that anti-IGFBP7 therapy holds great promise to improve the response to immune therapy, making it potentially an excellent drug target for combination treatment with immunotherapy for BLCA, which certainly necessitates further studies to verify our speculations.

## Data Availability Statement

The datasets presented in this study can be found in online repositories. The names of the repository/repositories and accession number(s) can be found in the article/**Supplementary Material**.

## Ethics Statement

Our study did not require an ethical board approval because this article does not contain any studies with human participants or animals performed by any of the authors.

## Author Contributions

JA, XL and XY conceived the project and drafted the manuscript, XZ, HX, JL, DL, GP and TZ collected the public data and performed the bioinformatics analysis. HL and JA revised the manuscript. XL made great contributions during the revision of the manuscript. All authors contributed to the article and approved the submitted version.

## Funding

This study was supported by grants from the National Natural Science Foundation of China (82070784,81702536) to J. A., a grant from Science & Technology Department of Sichuan Province, China (2022JDRC0040) to J. A., a grant from the National Natural Science Foundation of China (81871567) to X. L.

## Conflict of Interests

The authors declare that the research was conducted in the absence of any commercial or financial relationships that could be construed as a potential conflict of interest.

## Publisher’s Note

All claims expressed in this article are solely those of the authors and do not necessarily represent those of their affiliated organizations, or those of the publisher, the editors and the reviewers. Any product that may be evaluated in this article, or claim that may be made by its manufacturer, is not guaranteed or endorsed by the publisher.
