# Supplementary material for: IGFBP7 and the Tumor Immune Landscape: A Novel Target for Immunotherapy in Bladder Cancer
Source: Front Immunol. 2022 Jun 23;13:898493. doi: 10.3389/fimmu.2022.898493 (PMC9259832; doi:10.3389/fimmu.2022.898493)

**Table S1** **Abbreviations of cancers in TCGA**

| Abbreviation | Cancer type |
| --- | --- |
| ACC | Adrenocortical carcinoma |
| BLCA | Bladder urothelial carcinoma |
| BRCA | Breast invasive carcinoma |
| CESC | Cervical squamous cell carcinoma and endocervical adenocarcinoma |
| CHOL | Cholangiocarcinoma |
| COAD | Colon adenocarcinoma |
| COADREAD | Colon adenocarcinoma/Rectum adenocarcinoma Esophageal carcinoma |
| DLBC | Lymphoid neoplasm diffuse large B-cell lymphoma |
| ESCA | Esophageal carcinoma |
| GBM | Glioblastoma multiforme |
| GBMLGG | Glioma |
| HNSC | Head and neck squamous cell carcinoma |
| KICH | Kidney chromophobe |
| KIRC | Kidney renal clear cell carcinoma |
| KIRP | Kidney renal papillary cell carcinoma |
| LGG | Brain lower grade glioma |
| LIHC | Liver hepatocellular carcinoma |
| LUAD | Lung adenocarcinoma |
| LUSC | Lung squamous cell carcinoma |
| MESO | Mesothelioma |
| OV | Ovarian serous cystadenocarcinoma |
| PAAD | Pancreatic adenocarcinoma |
| PCPG | Pheochromocytoma and paraganglioma |
| PRAD | Prostate adenocarcinoma |
| READ | Rectum adenocarcinoma |
| SARC | Sarcoma |
| SKCM | Skin cutaneous melanoma |
| STAD | Stomach adenocarcinoma |
| TGCT | Testicular germ cell tumors |
| THCA | Thyroid carcinoma |
| THYM | Thymoma |
| UCS | Uterine carcinosarcoma |
| UVM | Uveal melanoma |

**Supplementary Figure S1.** **Correlation between IGFBP7 and immune checkpoints.**


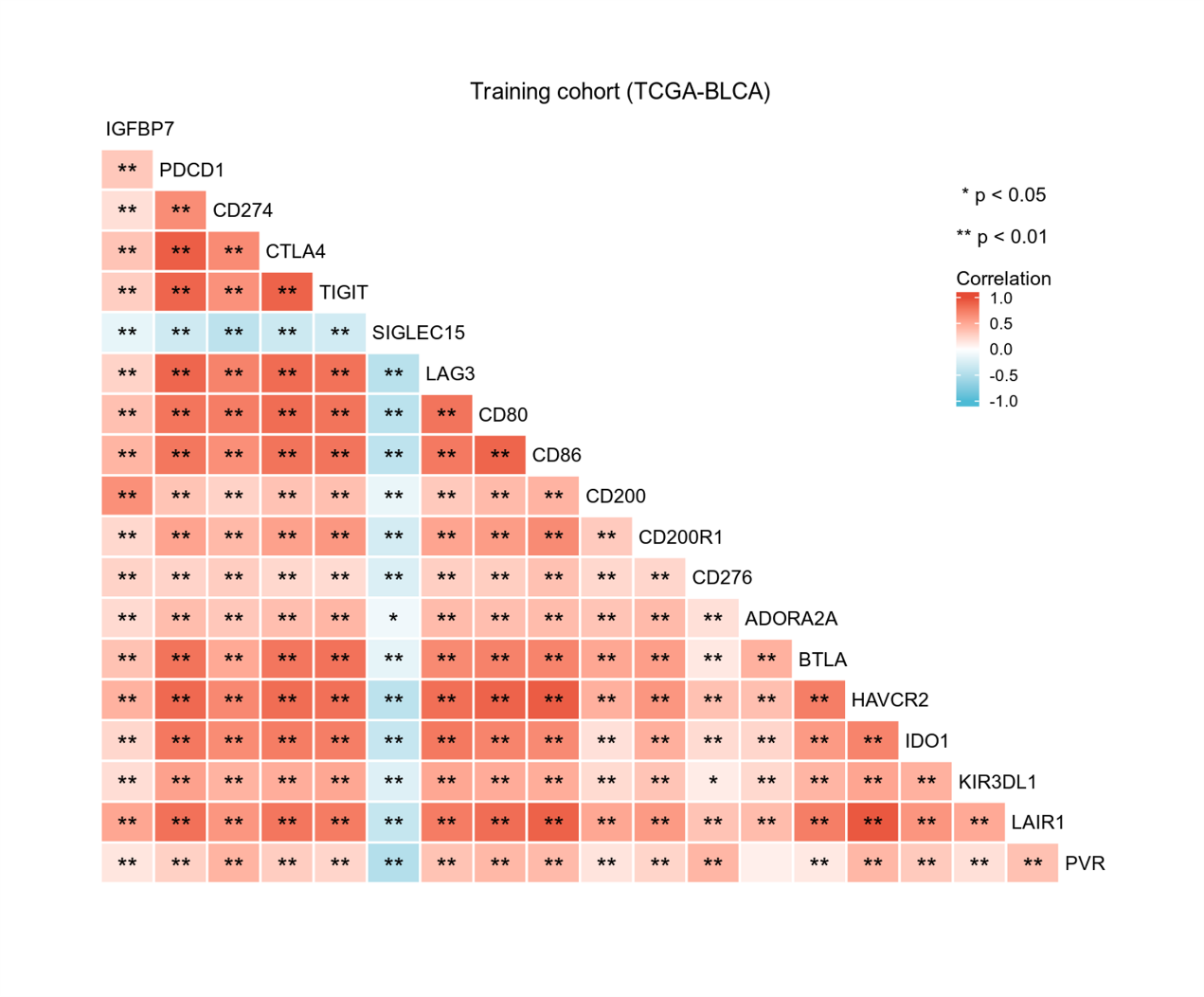


**Supplementary Figure S2.** (A-D) Differences in the enrichment scores of 12 molecular subtype-specific signature between the high- and low-IGFBP7 groups using validation cohorts: IMvigor210 and GSE176307. (E-H) Validation of the value of prediction therapeutic response to several therapies in BLCA by IGFBP7. (I, J) Correlation between IGFBP7 and the BLCA-related drug-target genes obtained from the Drugbank database.


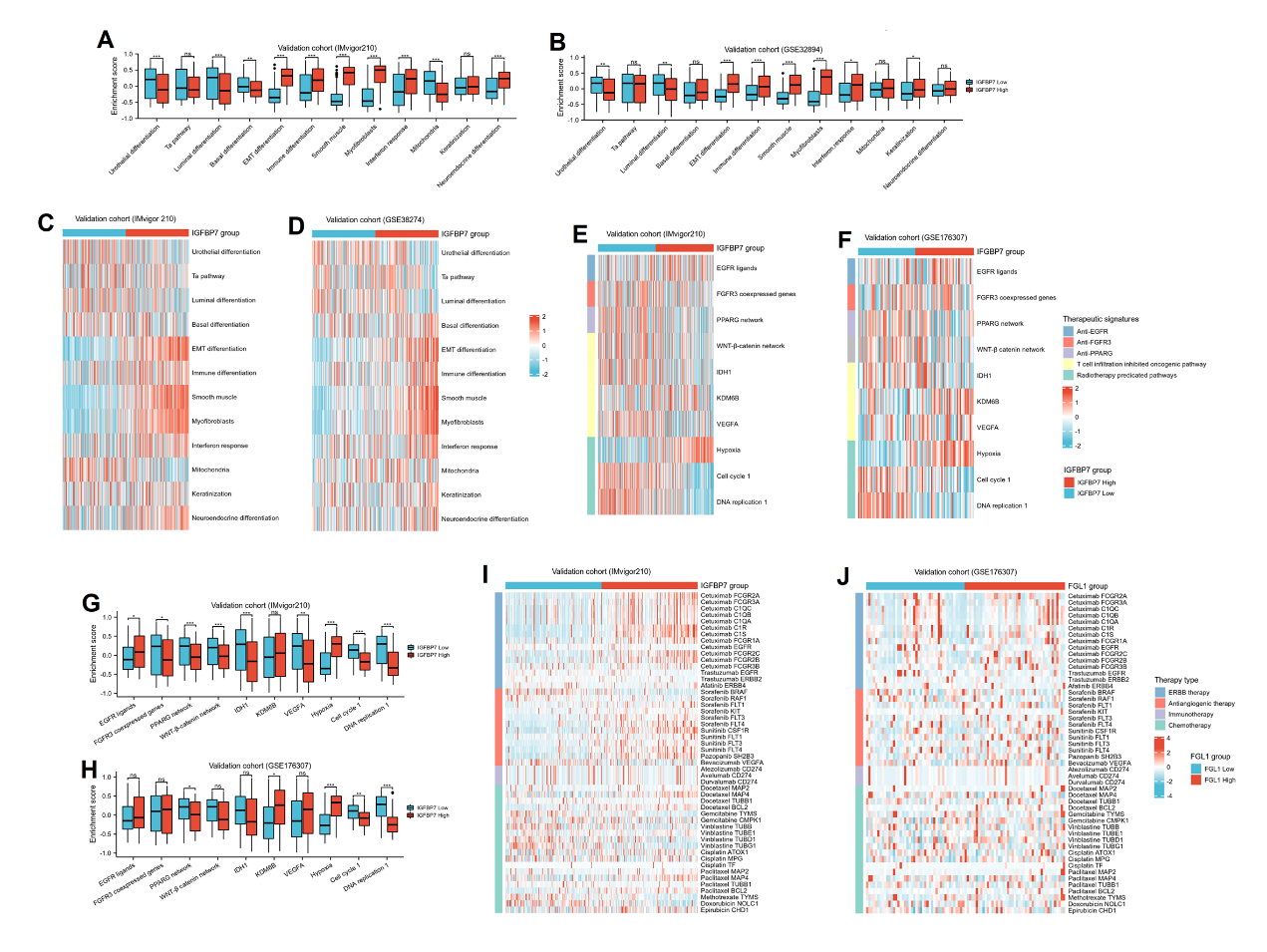


**Supplementary Figure S3.** (A) waterfall plot of mutation data of the top30 mutation gene. (B) Differences in tumor mutation burden between High and IGFBP7 Low groups.


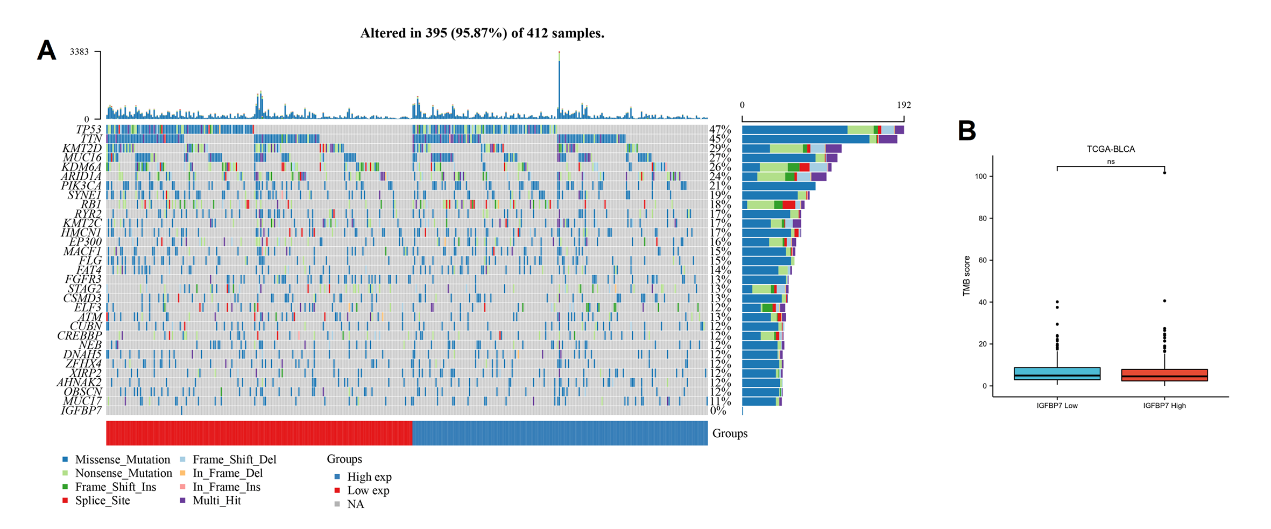


**Supplementary Figure S4. Validation of IGFBP7-based immune risk model by TCGA cohort.**


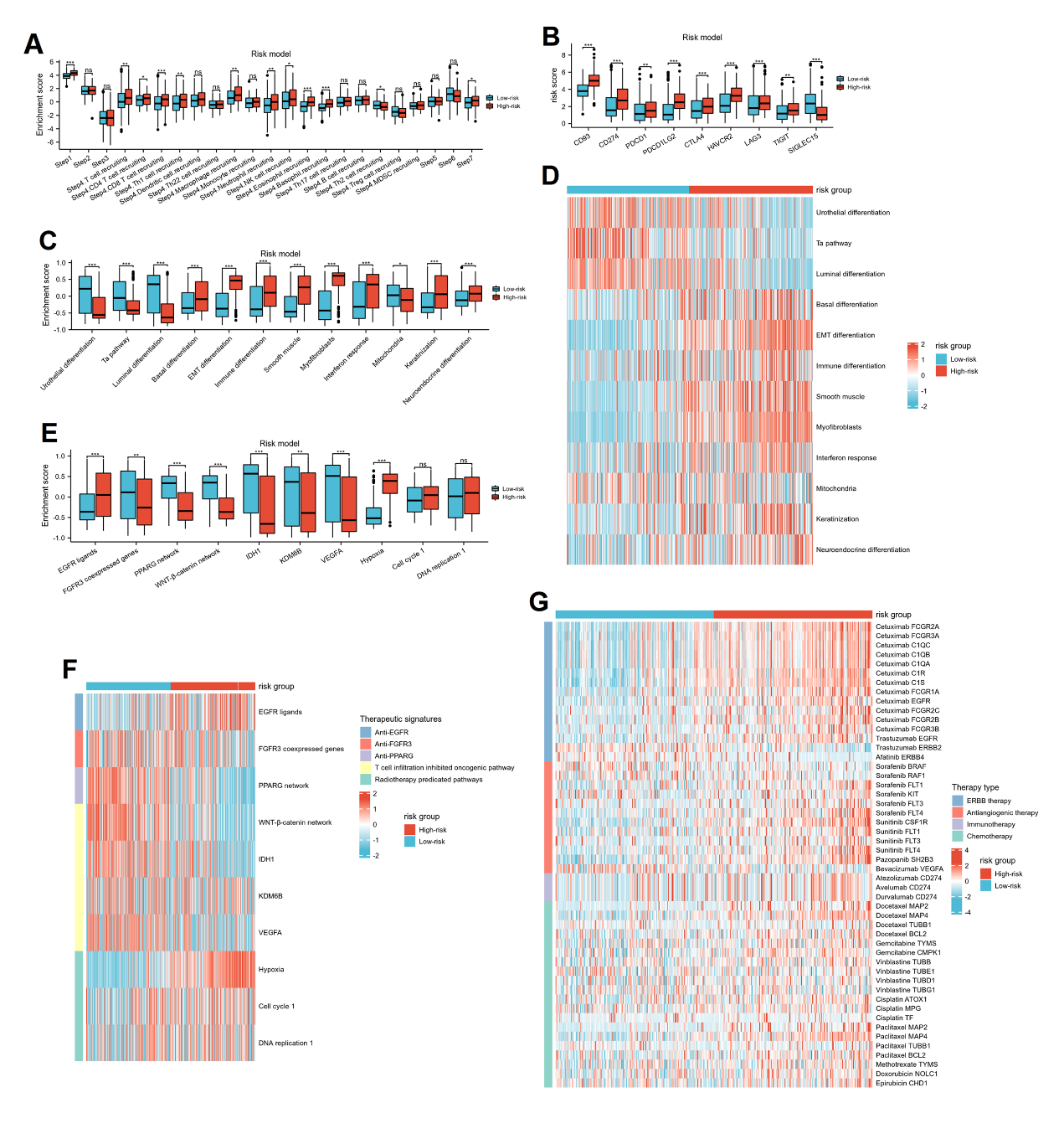

Supplement: Supplementary file 1 [file DataSheet_1.docx]
